# Integrating sensory evaluation into biofortification breeding: assessing consumer acceptance and market segmentation of porridge made from biofortified pearl millet cultivars

**DOI:** 10.3389/fnut.2025.1594589

**Published:** 2025-08-26

**Authors:** Manhal Gobara Hamid, Mohammed Elsafy, Tilal Abdelhalim

**Affiliations:** ^1^Biotechnology and Biosafety Research Center, Agricultural Research Corporation, Khartoum North, Sudan; ^2^College of Agricultural Studies, Sudan University of Science and Technology, Khartoum North, Sudan; ^3^Department of Plant Breeding, Swedish University of Agricultural Sciences (SLU), Alnarp, Sweden

**Keywords:** consumer acceptance, market segmentation, sensory profiling, biofortified foods, pearl millet

## Abstract

**Introduction:**

Sensory evaluation is a crucial tool in biofortified pearl millet breeding, influencing consumer acceptance and market expansion. This study aimed to evaluate the sensory attributes and consumer acceptability of stiff porridge (*Aceda*) prepared from biofortified pearl millet and its blends with traditional Sudanese cultivars to inform breeding and market strategies.

**Methods:**

This study was conducted in 2023 at the Gadarif Research Station, Agricultural Research Corporation (ARC), Sudan. It evaluated the sensory attributes of stiff porridge (*Aceda*) made from biofortified pearl millet (Aziz), two widely adopted Sudanese cultivars (Ashana and Bayoda), and their blended formulations. To analyze consumer segmentation and preference patterns, 28 semi-trained assessors conducted hedonic scoring and rapid descriptive profiling. The data were analyzed using internal preference mapping (IPM), panel analysis, product characterization, and partial least squares regression (PLSR) using XLSTAT.

**Results and discussion:**

The results revealed significant variation among the five *Aceda* products (analysis of variance (ANOVA), *F* = 11.84, *p* < 0.001), with the Bayoda + Aziz blend receiving the highest liking score (mean = 7.7) and Aziz alone the lowest (mean = 5.8). Principal component analysis (PCA) explained 71.47% of total variation in consumer preference, while PLSR identified taste, firmness, aroma, and texture as key drivers of acceptance. Panel analysis revealed that firmness (*F* = 13.22, *p* < 0.0001), color, and appearance exhibited the highest discriminative power among the descriptors. Short-term recommendations include blending biofortified cultivars with preferred local varieties to enhance adoption. Long-term strategies should integrate omics-enabled breeding with sensory and market-oriented selection.

**Conclusion:**

This study provides the first quantitative evidence of consumer sensory preferences for biofortified *Aceda*, emphasizing that blending strategies significantly enhance acceptability. Integrating sensory evaluation into early stage breeding, alongside artificial intelligence (AI) and rapid sensory tools, will accelerate the adoption of biofortification and support nutrition-sensitive breeding.

## 1 Introduction

Pearl millet [*Pennisetum glaucum* (L.) R. Br.] is an underutilized, climate-resilient, and drought-tolerant crop with enormous nutritional and yield potential in marginal lands (1). This crop is a staple food for nearly 90 million people living in poverty. It is extensively cultivated on 30 million hectares in the dry and semi-arid tropical regions of Asia and Africa, accounting for over half of the world’s millet production (2).

Pearl millet is gluten-free and has a low glycemic index; thus, it is suitable for gluten allergy treatment (2, 3). The crop is rich in vitamins, minerals, and essential amino acids, making it a good source of energy, carbohydrates, and fats (4). It contributes to antioxidant activity and protects against cancer, cardiovascular disease, and age-related diseases (5). It provides 30–40% inorganic nutrients and is an affordable staple food with iron and zinc (6). Owing to its nutritional properties, pearl millet has been renamed a nutricereal (2). Overcoming malnutrition and ensuring food and nutritional security is vital.

Biofortification, the process of increasing the nutritional value of staple crops by harnessing the power of plant breeding and advanced omics techniques, has been acknowledged as a cost-effective and sustainable strategy for combating widespread malnutrition or anemia in communities that rely heavily on cereal-based diets (7). With the support of HarvestPlus, researchers at the International Crops Research Institute for the Semi-Arid Tropics (ICRISAT) developed Africa’s first iron-biofortified pearl millet cultivar “Chakti” using conventional plant breeding techniques (8). Chakti is a high-yielding, early maturing, drought-tolerant pearl millet variety with grain iron (60 ppm) and zinc (45 ppm) densities (9). In Sudan, Chakti was introduced by ICRISAT and officially released in 2022 under the name Aziz, which is a fortified Arabic name.

In addition, Neeraja et al. (10) reported that 250 g of biofortified pearl millet daily can meet 84% of the recommended dietary allowance (RDA) for iron and 100% of the RDA for zinc in pregnant and lactating women. In contrast, ordinary millet fulfills only 20% of its iron requirement. Govindaraj et al. (11) demonstrated that the absorption and bioavailability of iron in pearl millet are sufficient to fulfill > 50% of the daily requirement of adult males and children. Additionally, in a study, Kodkany et al. (12) found that 50–100% of the daily iron requirement can be met with one meal of biofortified high-iron pearl millet varieties, which is sufficient to overcome iron deficiency in children, women, and men.

However, the adoption of biofortified crops largely depends on consumer and producer willingness to accept newly bred crop varieties (13, 14). In a study, Talsma et al. (14) reported that producers adopted cultivars with higher yields, disease and insect resistance, and profitability. Consumers are concerned about the sensory scores of color, aroma, taste, texture, and overall acceptability of biofortified crops, which are essential factors for their adoption and can affect plant breeders (15). Interestingly, in a study by Pillay et al. (16), they found that yellow provitamin A-biofortified maize can successfully address vitamin A deficiency, particularly in preschool-aged children. However, this approach may not be successful in older groups unless other methods are implemented, such as intensive nutrition education programs, targeting market prices, increasing availability in local grocery stores, and improving sensory properties through breeding (13, 14, 17–19).

Despite their nutritional superiority, biofortified cultivars are often hindered by low sensory appeal. Studies have shown that nutritional value alone does not guarantee consumer acceptance, particularly when sensory attributes such as taste, aroma, texture, and visual appearance do not align with local preferences. Sensory traits, especially taste, firmness, and aroma, play a pivotal role in shaping consumer choices and must be prioritized in biofortification breeding programs. Neglecting these traits can limit adoption even when a variety delivers proven health benefits. Therefore, integrating sensory evaluation into breeding pipelines is essential for developing nutritionally enhanced cultivars that are also culturally acceptable and market-preferred.

Accordingly, multifaceted strategies are required to position biofortified pearl millet as a mainstream dietary choice for Sudanese communities, including sensory science, plant-breeding innovations, and targeted market segmentation. Sensory-based breeding, an integral part of biofortification programs, can enhance consumer adoption, improve dietary diversity, and contribute to global food security. However, few studies in Sudan have evaluated the sensory attributes of traditional food products made from biofortified cultivars or assessed the feasibility of their integration with preferred local varieties. This gap limits our understanding of how to guide sensory-driven breeding and market targeting.

Therefore, the objective of this study was to assess the sensory characteristics, consumer segmentation, and acceptability of *Aceda* porridge prepared using three Sudanese pearl millet cultivars and their blends with the biofortified cultivar Aziz, using hedonic scoring, rapid sensory profiling, and multivariate statistical modeling.

## 2 Materials and methods

### 2.1 Plant materials

This study used three pearl cultivars: two officially released pearl millet cultivars, Ashana and Aziz, and a Sudanese farmer-preferred cultivar., Bayoda. Ashana is a high-yielding, early maturing cultivar with a grey pericarp. It is particularly valuable because of its resistance to downy mildew, a significant disease of the pearl millet. Aziz is a biofortified pearl millet cultivar recently released because of its exceptional grain iron (60 ppm) and zinc (45 ppm) concentrations. It matures early and produces high yields. Aziz is Africa’s first iron biofortified pearl millet cultivar., initially at ICRISAT, India, through conventional intrapopulation breeding that focused on enhancing grain iron content. It was first released under the name “Chakti” before being introduced to Sudan. Bayoda is a farmer-preferred variety in Sudan known for its high yield, white seeds, and early maturity. It is commonly used to prepare porridge with milk, especially during the month of Ramadan. In addition, it exhibits resistance to the white group, a trait that enhances its popularity among Sudanese farmers, particularly in Western Sudan. Seeds of all three pearl millet cultivars were obtained from the Pearl Millet Breeding Program of the Agricultural Research Corporation (ARC), Wad Medani, Sudan.

The complete study, including porridge preparation and sensory testing, was conducted in February 2023 at the Gadareif Research Station of the Agricultural Research Corporation (ARC) in Sudan.

### 2.2 Porridge preparation

**Figure 1:**
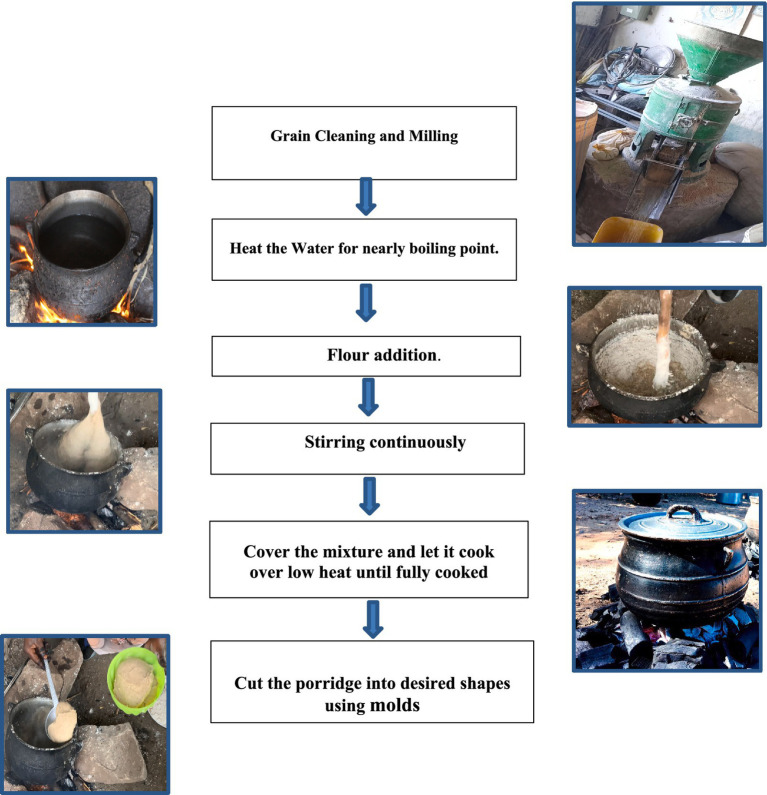
Flowchart illustrating the preparation steps of traditional stiff pearl millet porridge (*Aceda*), starting from grain cleaning and milling to shaping the final product. The process involves heating water, gradually adding flour, continuous stirring, cooking over low heat, and molding into the desired shapes.

For porridge preparation, the pearl millet grains were cleaned to remove any broken seeds or impurities before being ground using an electric commercial stone mill. The traditional stiff Sudanese porridge, *Aceda,* is prepared using a method commonly used by Sudanese women. Pearl millet flour was gradually added to boiling water at a flour-to-water ratio of 1:2 (w/v) with continuous stirring using a wooden spatula. The mixture was stirred until a smooth, uniform texture was obtained. Porridge was then boiled for 5 min to thicken the dough and develop the characteristic stiffness of *Aceda* (Figure 1). The particular flour blend ratio used for porridge preparation was chosen based on preliminary trials and local culinary practices to achieve an optimal balance between texture, firmness, and ease of stirring. The five porridge samples were maintained at room temperature (27 ± 2°C) and covered with aluminum foil in transparent plastic plates. The samples were subsequently used for hedonic and rapid sensory profiling.

### 2.3 Ethical statement

This study was reviewed and approved by the Nutrition Ethics Committee of the Federal Ministry of Health, Sudan. Before participation, all panelists who were staff members at the Gadareif Research Station, Agricultural Research Corporation (ARC), Sudan, provided written informed consent. Each participant was thoroughly briefed on the study objectives and protocols, and had an opportunity to ask questions and express any concerns before making an informed decision to participate. All the participants signed and returned consent documents to the research team. Strict protocols were followed to protect the participants’ rights and privacy throughout the sensory evaluation. This study adhered to the ethical standards and regulations outlined in the Declaration of Helsinki.

### 2.4 Participants, training sessions, and descriptor development

This study involved 28 assessors, comprising 15 women and 13 men, aged 25–60 years. The assessors were selected based on their frequency of porridge consumption, availability, health status, absence of allergies, and overall suitability, which was determined through personality assessment. All assessors were staff members at the Gadareif Research Station of the Agricultural Research Corporation (ARC) in Sudan.

A structured four-hour training session was conducted at the conference hall of the Gadareif Research Station (ARC) to enhance sensory evaluation skills. The training provided a theoretical foundation for sensory descriptions, enabling assessors to measure, recognize, and describe key sensory descriptors. The session began with an introduction to sensory vocabulary and evaluation approaches, including hedonic scales, rapid descriptor analysis, and data collection methods, as outlined in a study by Silva et al. (20). Assessors were then trained to detect and assess various sensory descriptors, including taste, firmness, cohesiveness, aroma, aftertaste, texture, and mouthfeel, using triangular and ordering tests (21). To facilitate this, each assessor received five porridge samples during the training session (22).

Training was conducted in two separate sessions, each lasting approximately 2 h. Through consensus, the assessors developed sensory descriptors and definitions to capture the differences among the five porridge samples, which included three pearl millet cultivars and two blends incorporating the biofortified Aziz cultivar., in accordance with the guidelines recommended in a study by Maraval et al. (23). The group also established standardized anchor terms and selected reference standards for evaluation (22). By the end of the training, nine sensory descriptors/attributes had been identified: appearance, color, aroma, cohesiveness, firmness, taste, mouthfeel, texture, and overall acceptability.

### 2.5 Sensory evaluation

#### 2.5.1 Hedonic test

Five *Aceda* products were prepared using three pearl millet cultivars and two blends with the biofortified cultivar Aziz, and served on coded plates for hedonic evaluation. Each sample was assigned a unique three-digit random code to eliminate bias and to ensure precise identification. The hedonic assessment was conducted in a single session by 28 semi-trained assessors. Before the evaluation, each assessor was given a brief introduction to the 9-point hedonic scale to ensure consistency in scoring. The overall liking and hedonic scores were collected using a 9-point hedonic scale (1 = extremely dislike to 9 = extremely like), following the method described in a study by Nicolas et al. (24). During the evaluation, the assessors were instructed to cleanse their palates with water between samples and prevent residual taste interference.

The testing environment was carefully controlled to maintain optimal sensory conditions, with a room temperature of 27 ± 2°C and a relative humidity between 40 and 50%. The panelists were seated individually, with sufficient spacing to prevent interactions and discussions, thus ensuring an unbiased assessment. Each assessor was given a 5-min break after evaluating the three samples to minimize sensory fatigue and maintain concentration. Additionally, water and unsalted crackers were provided for palate cleansing between the *Aceda* samples to prevent carryover effects and ensure accurate evaluations.

#### 2.5.2 Rapid description sensory profiling

Rapid descriptive sensory profiling was conducted by 28 assessors who evaluated the intensity of the sensory descriptors of the five *Aceda* products. The descriptors were categorized according to appearance, color, aroma, cohesiveness, firmness, taste, mouthfeel, and texture as defined in the sensory lexicon. The assessors rated these attributes using a 9-point hedonic scale, following the method described in a study by Balthazar et al. (25), where 1 represented “dislike extremely” and 9 represented “like extremely.”

Each panelist was presented with five *Aceda* samples (weighing approximately 100 g) on transparent plastic plates. To prevent first-order carryover effects, the samples were labeled with unique three-digit random numbers and presented in a monadic sequential order, following a balanced complete block design outlined in a study by MacFie et al. (26). A 5-min break was provided between samples to minimize sensory fatigue and ensure accurate assessment. The testing environment was maintained under the same controlled conditions as in the hedonic analysis, guaranteeing consistency in temperature (27 ± 2°C) and relative humidity (40–50%). Each panelist was also given a 500-ml bottle of purified drinking water (Safia®) to cleanse their palate before and after testing each *Aceda* product and conducting unbiased evaluations.

### 2.6 Statistical analysis

The hedonic scores for the *Aceda* products were analyzed using one-way analysis of variance (ANOVA) to determine significant differences among the *Aceda* samples. Statistically significant differences (*p <* 0.05) were identified by Tukey’s multiple comparison test using XLSTAT (version 2025, Addinsoft, USA). Internal preference mapping (IPM) based on principal component analysis (PCA) was employed to analyze the assessor ratings of the five *Aceda* samples, following the approach described in a study by Fernández-Vázquez et al. (27). To segment a homogenous group of assessors based on overall liking, agglomerative hierarchical clustering (AHC) was performed using automatic entropy truncation based on entropy, as outlined in a study by Fliedel et al. (28).

Panel analysis was used to assess the ability of the 28 assessors to differentiate *Aceda* products using sensory lexicon descriptors and to determine the reliability of their ratings.

Product characterization was conducted using an ANOVA model to identify the descriptors that best discriminated among the *Aceda* products and determine the most defining characteristics of each sample, following the study by Esmerino et al. (29). We applied an ANOVA model for each descriptor to test whether assessor scores differed significantly. The model used was Y = product effect + assessor effect, where assessor effect was treated as random, whereas product was considered fixed. Factor.

A biplot with 95% confidence ellipses was developed to visualize the sensory profiles obtained from PCA, following the method developed in a study by Husson et al. (30). These confidence ellipses, whose orientation and surface were based on the scores given by different assessors, were calculated using a resampling method to visually represent the relationship between sensory descriptors and *Aceda* products.

Partial least squares regression (PLSR) was performed following the protocol recommended in a study by Tenenhaus et al. (31) to further explore the relationship between the sensory descriptors and product characteristics. This analysis identified *Aceda* as the product with the highest commercialization potential. Additionally, heatmap analysis using XLSTAT was conducted as described in a study by Garrido-Bañuelos et al. (32) to examine the correlation between descriptors and *Aceda* product samples. These were independently clustered using ascendant hierarchical clustering based on Euclidean distances, which were reflected in a permuted matrix. The corresponding color intensities represent the data values, providing an intuitive visual interpretation of the relationships between the descriptors and product samples.

## 3 Results

### 3.1 Hedonic score

**Figure 2:**
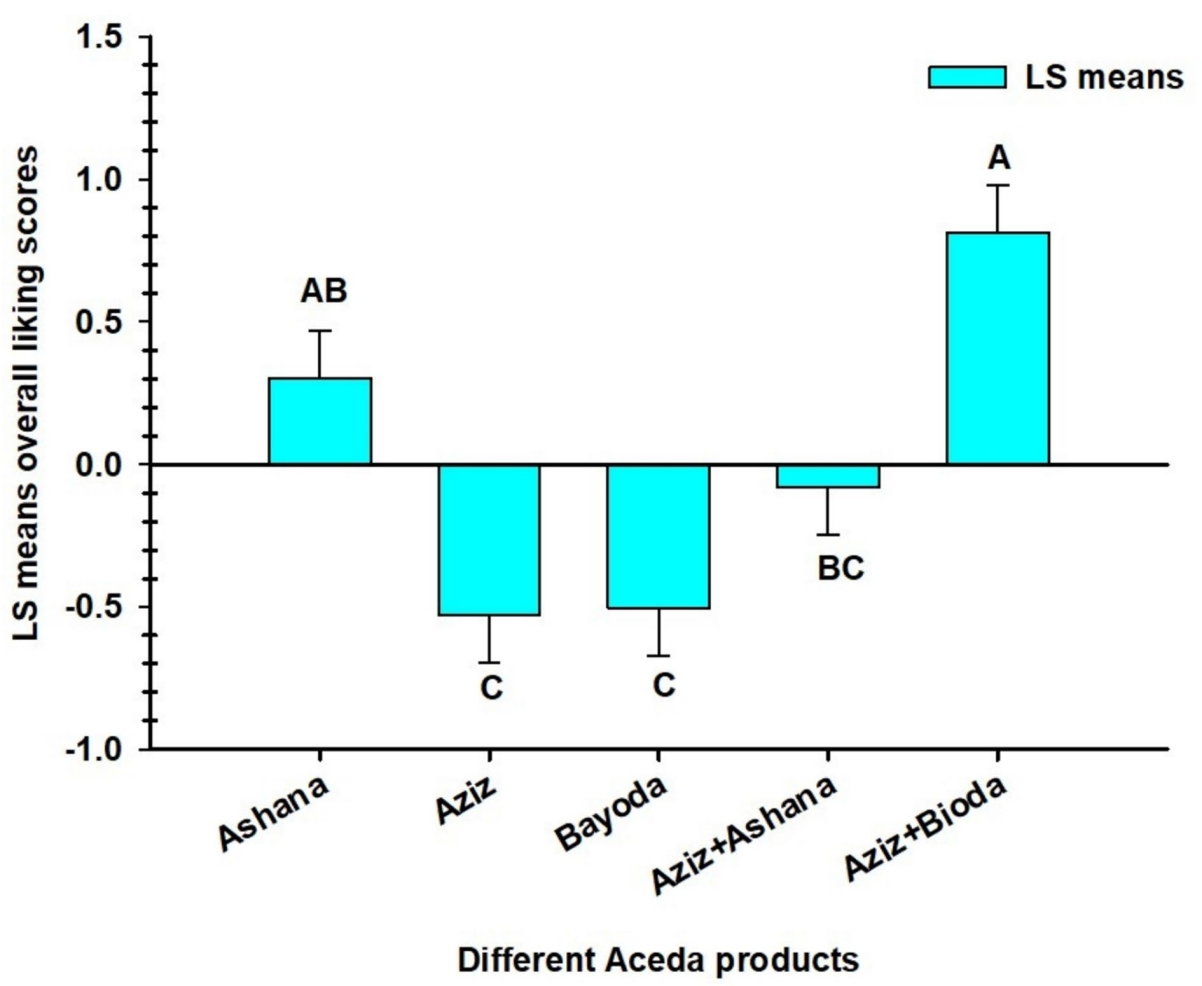
Least Squares (LS) means the liking scores for five *Aceda* products prepared from three pearl millet cultivars and their blends with the biofortified cultivar., Aziz. Bars represent the mean scores (*N* = 28) given by the assessors, with error bars indicating the standard errors of the means. Bars with different letters denote significant differences (*p* < 0.05) based on Tukey’s Honestly Significant Difference (HSD) test.

The results of the one-way ANOVA revealed highly significant variation (*p* < 0.001) among the five *Aceda* products (Table 1). The *Aceda* prepared from the Bayoda + Aziz blend received the highest preference score of 7.7, significantly higher than those of Bayoda, Aziz, and the Ashana + Aziz blend (Figure 2). However, no significant difference was observed between the Bayoda + Aziz blend and the Ashana alone. Similarly, no significant difference was detected between the Ashana + Aziz blend and the Ashana alone. The lowest preference score (approximately 5.8) was recorded for *Aceda,* made from the biofortified cultivar Aziz (Figure 2).

**Table 1:** One-way analysis of variance (ANOVA) results for five traditional stiff porridge (*Aceda*) products evaluated by 28 semi-trained assessors.

Source	DF	Sum of squares	Mean squares	F	Pr > F	*p*-values significance codes
Model	4	36.347	9.087	11.835	**<0.0001**	***
Error	135	103.653	0.768			
Corrected Total	139	140.000				

The model revealed highly significant differences among products (*p < 0.001). Computed against model Y = Mean(Y). Signification codes: 0 < *** < 0.001 < ** < 0.01 < * < 0.05 <. < 0.1 <.

**Figure 3:**
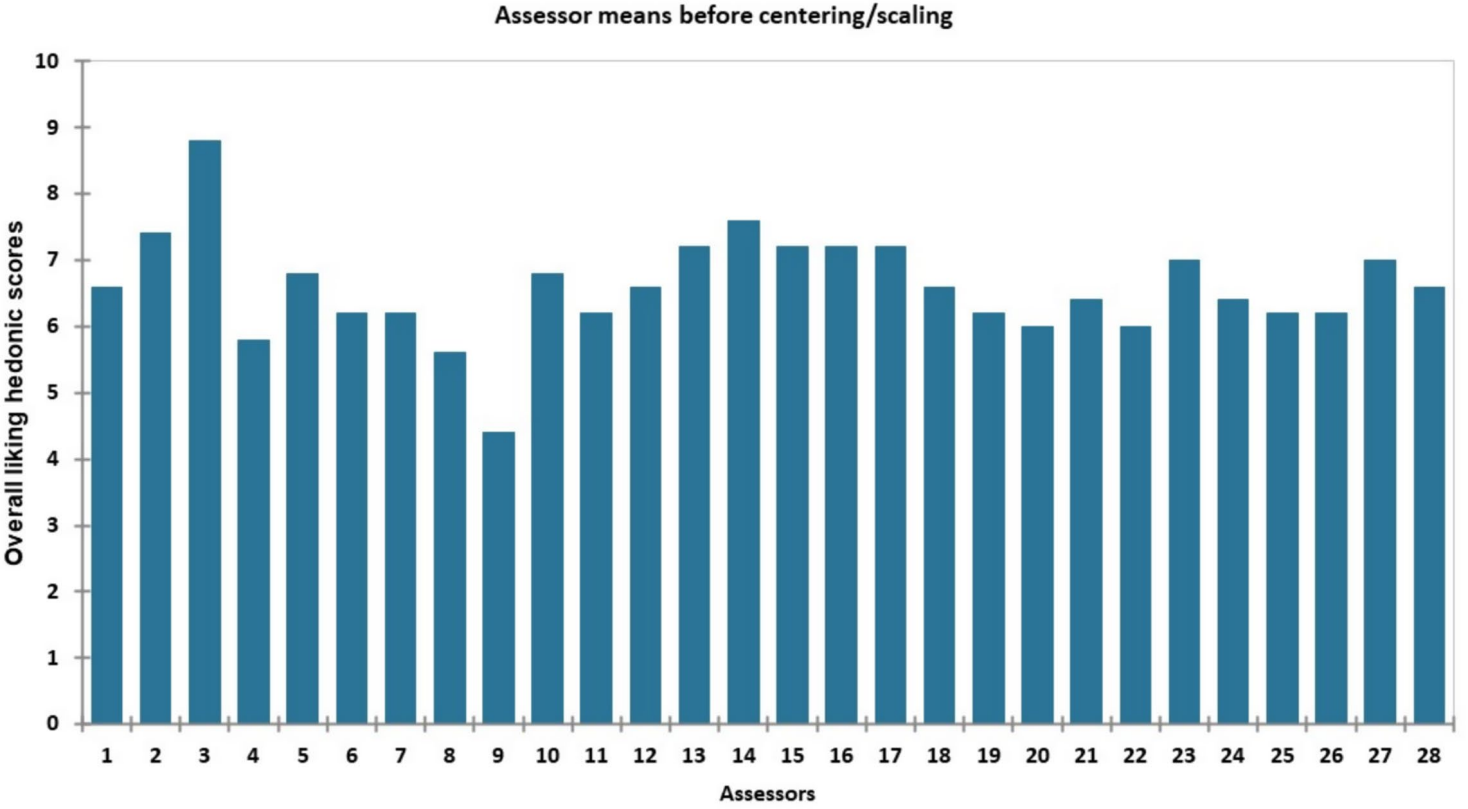
Mean overall liking hedonic scores for the five *Aceda* products provided by each of the 28 assessors before centering/scaling.

The mean hedonic scores for the five *Aceda* products as rated by the 28 assessors ranged from 4.4 to 8.8, with an overall average of 6.59. The highest score (8.8) was recorded by assessor 3, and the lowest score (4.4) was given by assessor 9. Most assessors provided scores within a relatively narrow range of 6.0 to 7.6, indicating a generally positive response to *Aceda* products. However, assessors 4, 8, and 9 were identified as atypical, with ratings below 6.0 (Figure 3).

### 3.2 Internal preference mapping (IPM)

**Figure 4:**
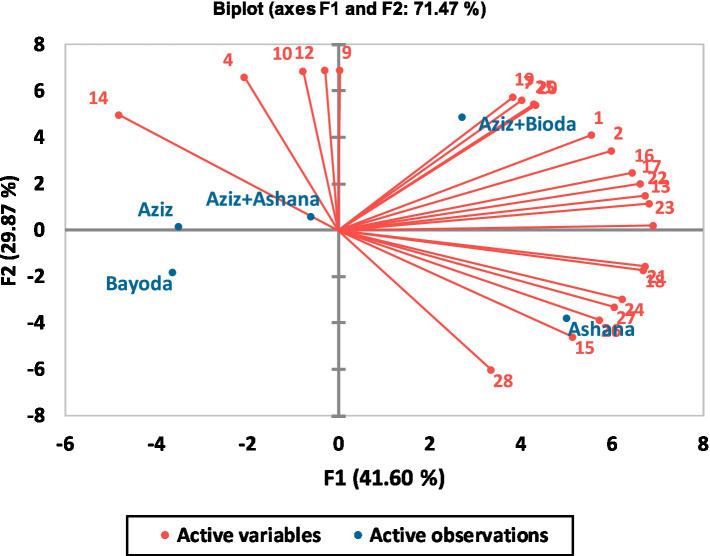
Internal preference mapping illustrates the relationship between the five *Aceda* products and the scores assigned by semi-trained assessors. The red vectors represent the individual assessors’ preferences, indicating the direction and strength of their ratings. Blue points correspond to the five *Aceda* products positioned within the preference space.

Internal preference mapping (IPM) based on PCA revealed that the first two principal components (F1 and F2) accounted for 71.47% of the total variation. F1 explained the most significant proportion of variability (41.60), while F2 contributed 29.87 (Figure 4). *Aceda* products prepared from Ashana, Aziz, and Bayoda were primarily associated with F1, whereas Bayoda and Aziz were strongly linked to F2. The Ashana + Aziz blend was positively associated with F4, indicating different underlying variability.

The positioning of the *Aceda* products within the preference space varied, reflecting distinct sensory preferences. The *Aceda* made from Ashana was positioned in the lower right quadrant, showing a strong association with F1 (r = 0.628), and was preferred by assessors 21, 18, 24, 27, 26, 15, and 28. The Bayoda + Aziz blend was located in the upper right quadrant and showed a high correlation with F2 (0.709), indicating a higher preference among multiple assessors.

Conversely, the *Aceda* prepared by Bayoda was positioned on the negative side of the lower left quadrant. At the same time, Aziz was located in the upper left quadrant, indicating lower preference levels among the assessors. Ashana + Aziz appeared near the center of the biplot, suggesting a balanced perception across the assessors, with no strong inclination toward preference or rejection.

### 3.3 Segmentation of assessors

**Figure 5:**
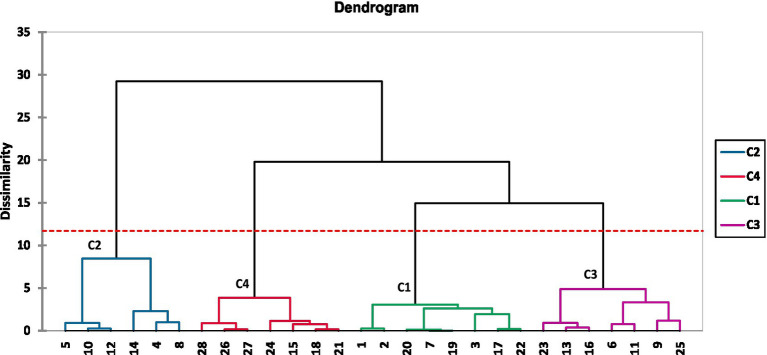
Hierarchical clustering of the 28 assessors based on their overall liking scores of the five *Aceda* products. Four distinct clusters (C1–C4) were identified, as indicated by the colored branches, using agglomerative hierarchical clustering with Euclidean distance and Ward’s linkage method. The red dashed line denotes the cut-off level used to define the clusters.

The agglomerative hierarchical analysis (AHC) dendrogram grouped the 28 assessors into four main clusters based on their overall liking scores for the five *Aceda* products (Figure 5). Cluster 3 had the highest number of assessors (8), while cluster 2 and cluster 4 had equal numbers (7 assessors each). The lowest number of assessors (6) was recorded in cluster 1. From the dendrogram, clusters 1 and 3 showed higher similarity, merging at a lower dissimilarity level. However, cluster 2 was the most distinct, merging at a higher dissimilarity level, indicating an extreme or unique preference pattern that sets it apart from the other clusters. Cluster 4 was positioned between cluster 2 and cluster 2, indicating moderate preference alignment with elements of both clusters (Figure 5).

**Figure 6:**
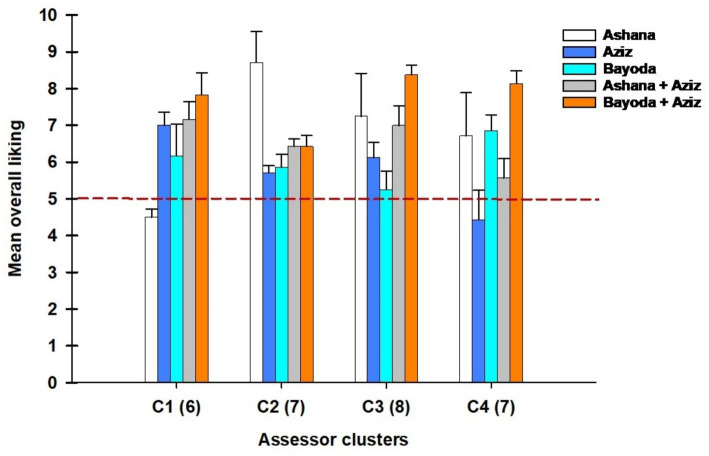
Mean overall liking scores for the five *Aceda* products across four assessor clusters (C1–C4). Error bars indicate the standard error of the mean (SEM) for each cluster-product combination. The red dashed line marks the neutral acceptability threshold at 5 points, distinguishing the rating of dislike (below 5) from those of preference (above 5). Numbers in parentheses indicate the number of assessors per cluster.

Figure 6 illustrates the relationship between the overall liking scores of *Aceda* products and assessor clusters. The red dashed line represents a score of 5, the threshold between general acceptance and rejection (neutral). Scores above the threshold indicate an *Aceda* product preference, whereas those at or below indicate a lack of preference. Clustering analysis revealed distinct assessor groups with specific inclinations toward the *Aceda* product.

Cluster 1 (C1) Assessors were identified as Ashana dislikers but strongly preferred the Ashana + Aziz and Bayoda + Aziz blends. By contrast, cluster 2 (C2) assessors were predominantly Ashana likers, demonstrating a clear preference for this product. Cluster 3 (C3) assessors favored blended products but disliked Aziz and Bayoda, indicating a unique pattern of product preference. In cluster 4 (C4), assessors were categorized as Aziz dislikers, but Bayoda + Aziz blend likers, suggesting a more substantial acceptance of the biofortified blends over individual cultivars. Notably, the Bayoda + Aziz blend emerged as the preferred *Aceda* product across multiple clusters, indicating its widespread acceptance compared to other *Aceda* products (Figure 6).

**Figure 7:**
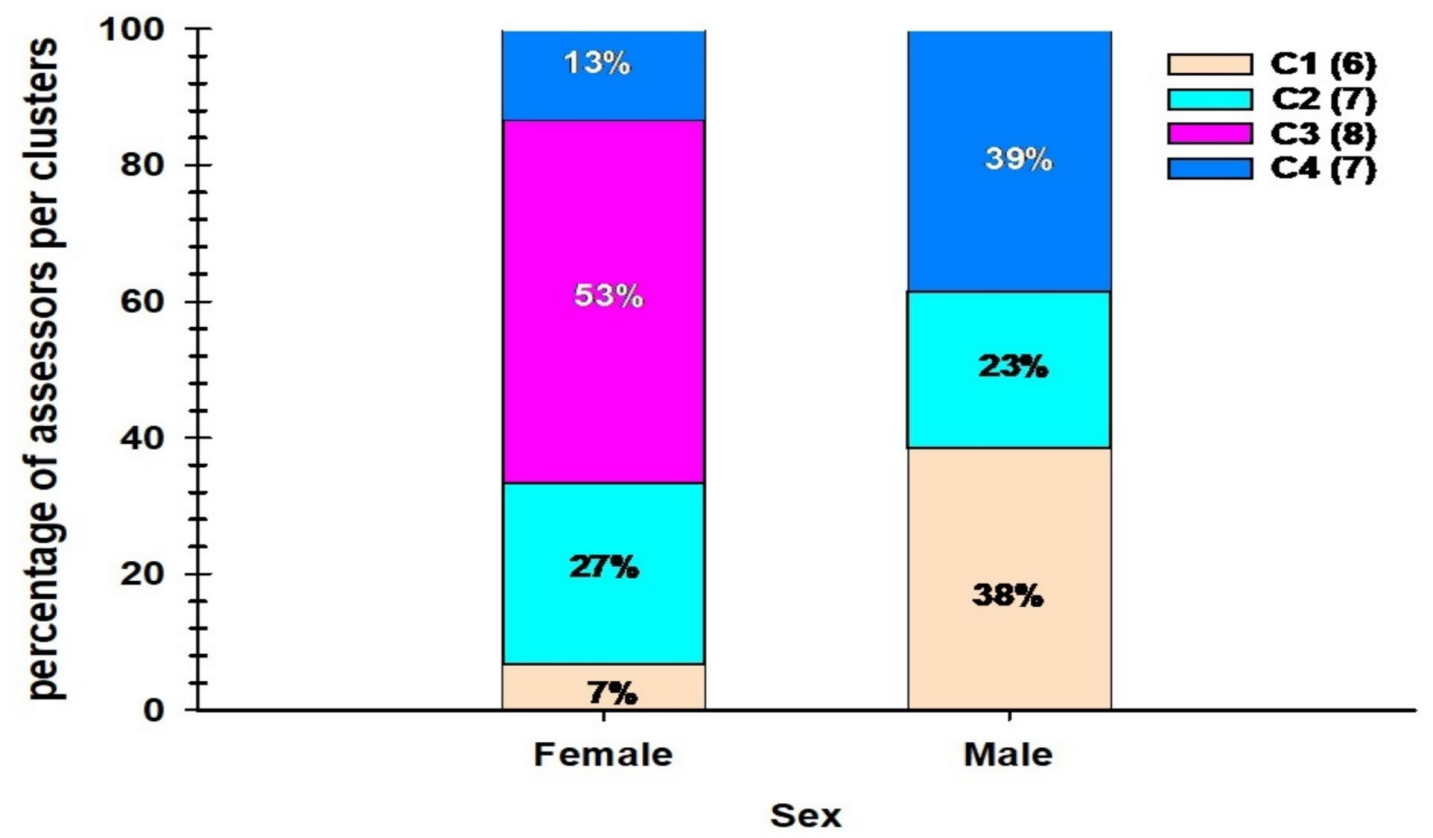
Percentage distribution of assessor clusters by sex. The stacked bar chart illustrates the proportion of male and female assessors assigned to each of the four sensory clusters (C1–C4) identified through hierarchical analysis. Values inside bars indicate the percentage of assessors per cluster within each gender group.

The distribution of assessors across the four clusters, based on gender, is illustrated in Figure 7, indicating differences in preference groupings between male and female participants. Among the female assessors, the most significant percentage (53%) of the total participating females belonged to cluster 3 (C3), followed by cluster 2 (C2) at 27%. A small proportion of female assessors (13%) were classified under cluster 3 (C3), whereas cluster 1 (C1) had the least representation, at only 7%.

For male assessors, cluster 4 (C4) accounted for the highest proportion (39%), followed by cluster 1 (C1) at 38%. cluster 2 (C2) had a smaller share of male assessors (23%), while cluster 3 (C3) was not represented among male assessors (Figure 7).

### 3.4 Panel analysis

The one-way ANOVA results revealed significant differences across all sensory descriptors evaluated by the assessors for *Aceda* products (Table 2). Firmness had the highest discriminative power (*F*-value = 13.22) in differentiating *Aceda* products, followed by color (F-value = 10.69), appearance (F-value = 9.26), and cohesiveness (F-value = 7.91), all of which were significantly different (*p* < 0.0001).

**Table 2:** Discriminating power of eight sensory descriptors for five *Aceda* products, as evaluated by 28 semi-trained assessors using ANOVA.

Descriptors	F	Pr > F
Firmness	13.22	<0.0001
Color	10.69	<0.0001
Appearance	9.26	<0.0001
Cohesiveness	7.91	<0.0001
Texture	4.89	0.001
Taste	4.16	0.004
Mouth feel	3.81	0.006
Aroma	3.26	0.015

The *F*-values and significance levels indicate how effectively each descriptor differentiated between products. Signification codes: 0 < *** < 0.001 < ** < 0.01 < * < 0.05 <. < 0.1 <.

Additionally, texture (F-value = 4.89, *p* = 0.001), taste (F-value = 4.16, *p* = 0.004), and mouthfeel (F-value = 3.81, *p* = 0.006) showed significant variation, albeit with moderate discriminative power. Aroma had the lowest discriminative power (F-value = 3.26, *p* = 0.0150), indicating a weaker role in differentiating products (Table 2).

**Figure 8:**
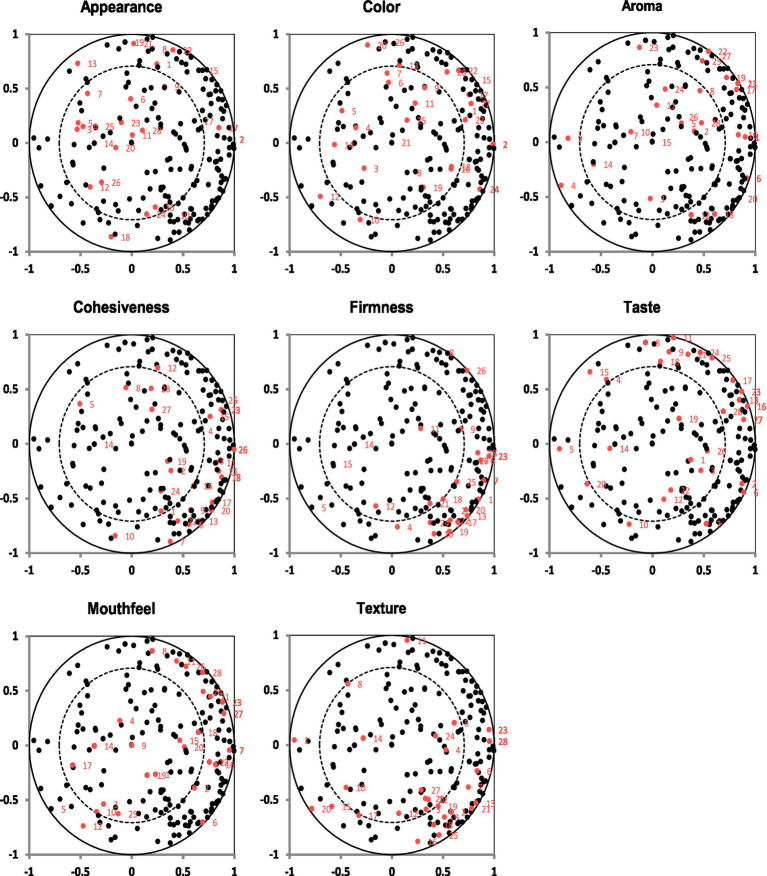
Principal component analysis (PCA) correlation plots for each sensory descriptor across assessors. Each subplot represents one descriptor: appearance, color, aroma, cohesiveness, firmness, taste, mouthfeel, and texture. Black dots represent all descriptor scores, while red dots highlight the 28 (assessor, descriptor) combinations, indicating the relationship and contribution of each assessor to the variability observed in the descriptor. The outer and inner circles denote 100 and 50% correlation limits, respectively.

A panel analysis conducted using XLSTAT evaluated the consensus among assessors in the sensory evaluations of various descriptors. The proximity of the red dots indicates a high level of agreement, suggesting that the designated descriptor was well understood and consistently rated. Conversely, a greater dispersion of red dots reflects variability in assessor ratings, indicating disagreement or difficulty in consistently evaluating an attribute (Figure 8).

For the appearance descriptor, the red dots were somewhat scattered, indicating moderate to low agreement among the 28 assessors. Similarly, the color descriptor showed moderate agreement, with some assessors providing similar ratings and forming small clusters (Figure 8). The aroma descriptors exhibited high dispersion, indicating significant variability in the assessments. In contrast, cohesiveness displayed a relatively high level of agreement, as indicated by the clustering of red dots, suggesting an overall consensus among the assessors (Figure 8).

For the firmness descriptor, the red dots demonstrate an acceptable level of agreement, reflecting a strong consensus in the evaluator ratings (Figure 8). The taste descriptor showed moderate dispersion, indicating fair agreement among the assessors. However, for mouthfeel, the high dispersion of red dots indicated significant variability in how assessors perceived and rated this attribute. Finally, the texture descriptor exhibited moderate agreement, as indicated by the clustering of red dots within the black dots, signifying a reasonable level of consensus among assessors (Figure 8).

### 3.5 Product characterization

Among the five *Aceda* products, Ashana received the highest scores for cohesiveness (7.750), taste (7.679), aroma (7.321), firmness (7.286), mouthfeel (7.101), and texture (7.000), indicating a strong preference for texture. However, it scored negatively for color (6.000) and appearance (6.286), indicating a need for improvement in visual aspects (Table 3).

**Table 3:** Adjusted mean sensory scores for eight descriptors across five *Aceda* products, as evaluated by 28 semi-trained assessors.

Product	Mouth feel	Aroma	Taste	Firmness	Texture	Cohesiveness	Color	Appearance
Ashana	7.107	7.321	7.679	7.286	7.000	7.750	6.000	6.286
Aziz	6.571	6.571	6.964	5.393	6.357	5.179	6.036	6.964
Bayoda	6.786	7.143	7.357	5.500	6.036	5.893	8.000	8.107
Ashana + Aziz	6.821	6.500	6.464	7.464	7.393	7.036	6.286	6.679
Bayoda + Aziz	7.964	7.821	8.071	8.214	7.607	6.929	8.036	8.071

Each product was rated for mouthfeel, aroma, taste, firmness, texture, cohesiveness, color, and appearance. Sensory evaluation of different *Aceda* product variations revealed distinct preferences among assessors, with blue-marked attributes indicating high ratings and red-marked attributes representing lower scores.

*Aceda* prepared from the biofortified pearl millet cultivar Aziz exhibited lower sensory ratings across multiple descriptors, particularly firmness (5.393) and cohesiveness (6.357), indicating a less favorable sensory profile. Bayoda excelled in color (8.000) and appearance (8.107), with moderate ratings for taste (7.679) and aroma (7.143), respectively. However, it was rated lower in terms of firmness (5.500) and cohesiveness (5.893), reflecting a trade-off between visual appeal and textural quality (Table 3).

The Ashana + Aziz blend showed improved firmness (7.464), texture (7.393), and cohesiveness (7.036) but received moderate scores for other descriptors. Among all the products, the Bayoda + Aziz blend received the highest ratings across key sensory attributes, including mouthfeel (7.964), aroma (7.821), taste (8.071), firmness (8.214), texture (7.607), and appearance (8.071), making it the most preferred variation (Table 3).

**Figure 9:**
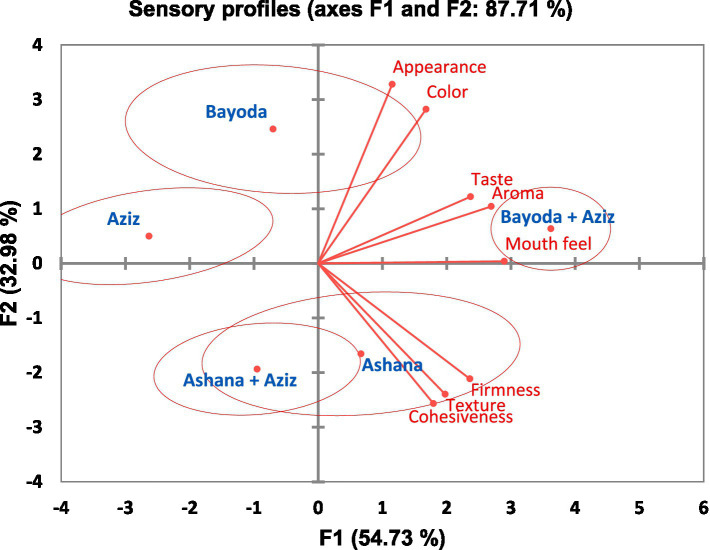
Principal component analysis (PCA) biplot illustrating the relationships between the five *Aceda* products and eight sensory descriptors. Products are represented as blue dots, while red vectors represent the sensory descriptors. The proximity and direction of each vector indicate the strength and nature of the correlation with the principal components. Product clusters are enclosed in red ellipses.

The biplot with 95% confidence ellipses generated from PCA classified the five *Aceda* products into four groups. Ashana and its blend with Aziz formed a single cluster, whereas the remaining three products were grouped separately. The first two principal components (F1 and F2) explained 87.71% of the total variation, with F1 accounting for 54.73% (eigenvalue = 4.378) and F2 explaining 32.98% (eigenvalue = 2.638) (Figure 9).

*Aceda* prepared from Aziz and the Bayoda + Aziz blend had the highest squared cosine values, indicating a strong association with F1. Bayoda was positively correlated with appearance and color, highlighting a strong visual appeal. However, the Ashana and Ashana + Aziz blends were linked to superior textural attributes, particularly firmness, texture, and cohesiveness. The Bayoda + Aziz blend, which was the most preferred, was closely associated with taste, aroma, and mouthfeel, thus reinforcing its superior sensory profile. In contrast, *Aceda* prepared from Aziz alone showed no strong preference across descriptors, indicating a lower overall acceptance (Figure 9).

### 3.6 Partial least squares regression (PLSR) and VIPs

**Figure 10:**
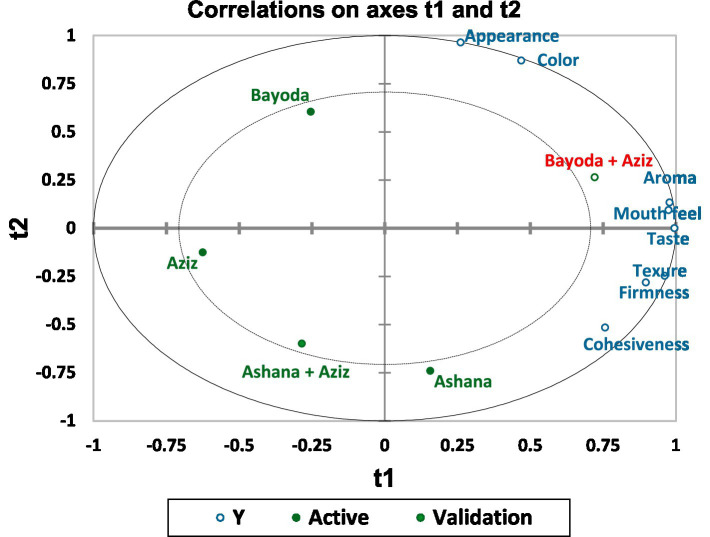
Correlation plot derived from Partial Least Squares regression (PLSR) illustrating the relationships between five *Aceda* products (green dots) and eight sensory descriptors (blue labels). The 95% confidence ellipse (outer solid line) and the Hotelling’s T^2^ ellipse (dotted line) indicate the precision and statistical confidence of the sample positions in the model.

The correlation plot in Figure 10 illustrates the distinct sensory profiles of the five *Aceda* products. According to the PLSR model, the Bayoda + Aziz blend, marked in red, has emerged as the most promising pearl millet *Aceda* product. This blend demonstrates a significant potential for broader adoption and market expansion. The value for the Bayoda + Aziz blend was strongly associated with textural and mouthfeel-related descriptors (Figure 10).

**Figure 11:**
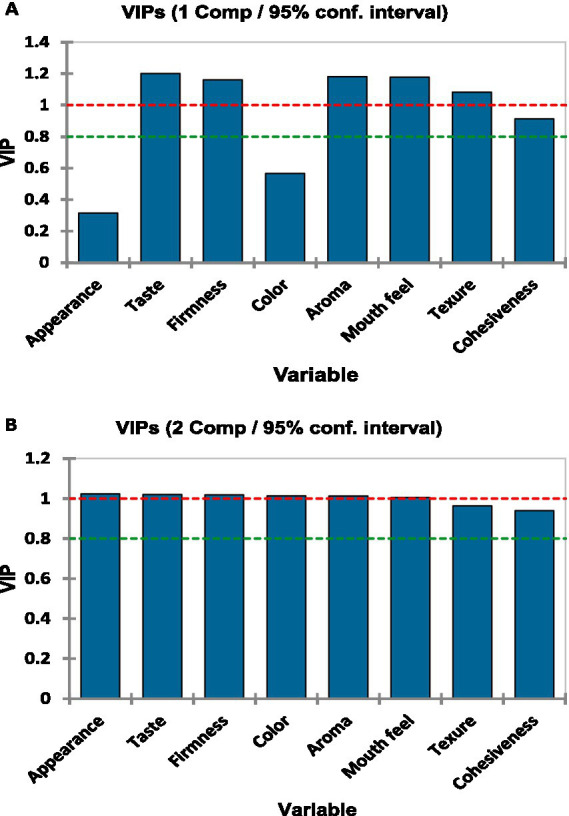
Variable importance in projection (VIP) scores from the partial least squares regression (PLS) model for the eight sensory descriptors across the first two components. **(A)** VIP scores for component 1 and **(B)** VIP scores for component 2. The red dashed line indicates the standard threshold of VIP = 1.0, above which descriptors are considered highly influential in the model. The green dashed line marks the average VIP score across descriptors. Variable importance in the projection (VIP) scores for each descriptor for components 1 [see **(A)**] and 2 [see **(B)**].

Figure 11 displays the variable importance in projection (VIP) scores, highlighting the contribution of each variable to the model. In component 1, appearance, color, and cohesiveness had VIP values below the threshold of 1.0, indicating lower influence on the model. In contrast, taste, firmness, aroma, mouthfeel, and texture exhibited higher VIP scores, suggesting a more significant contribution (Figure 11A).

For component 2, appearance, taste, firmness, color, aroma, and mouthfeel had VIP values close to or above the threshold of 1.0, thereby emphasizing their strong influence on the model. Conversely, texture and cohesiveness contributed less because their VIP values remained below the threshold (Figure 11B).

## 4 Discussion

This study evaluated the sensory profile of *Aceda* prepared from biofortified pearl millet cultivar Aziz and two widely adopted Sudanese cultivars, Ashana and Bayoda. To enhance consumer acceptance while preserving the familiar taste of traditional varieties, flour blends (1:1 ratio) combining Aziz with Ashana and Bayoda were introduced. Sensory evaluation was conducted by semi-trained assessors (aged 25–60, male and female) using overall hedonic liking scores and rapid sensory profiling to assess the five *Aceda* products. This is the first study to compare *Aceda* made from biofortified and non-biofortified pearl millet cultivars using sensory parameters.

Our hedonic score analysis (Figure 2) revealed significant variations in consumer preferences across the *Aceda* products. *Aceda,* made solely from Aziz, received the lowest preference score, indicating that nutritional enhancement alone is insufficient for market adoption. This aligns with the findings discussed in a study by Govender et al. (33), where they reported that color stigma is a significant barrier to the consumption of carotenoid-rich biofortified sweet potatoes and maize. Similarly, a study by Beswa et al. (34) highlighted the need to improve sensory appeal in stiff porridge made from biofortified yellow maize despite its nutritional value.

In a survey reported in a study by De Groote and Kimenju (35) in Nairobi, Kenya, white maize remained the most preferred, with consumers requiring a 37% discount to accept biofortified yellow maize. Socioeconomic factors, including gender, education, and income, have a significant influence on consumer choices. Women preferred white maize, while higher education levels reinforced their preference for it. Additionally, income levels affect willingness to pay (WTP), with higher-income consumers showing lower demand for biofortified maize and greater price sensitivity.

Despite the proven nutritional value of biofortified cultivars, sensory acceptability remains a critical bottleneck for mainstream adoption. Sensory evaluation is often underutilized in early-generation testing of biofortified breeding lines, primarily due to cost, scale, and throughput limitations. However, incorporating rapid, low-cost sensory screening protocols in early-stage breeding pipelines, especially for large populations, can help eliminate lines with poor sensory potential before advancing to later stages.

To promote the acceptance of *Aceda* made from biofortified pearl millet, we propose a three-phase strategic approach: short-term, prioritizing agriculture-nutrition education and extension programs in rural Sudan, where malnutrition is prevalent and pearl millet is a staple. Okello et al. (36) showed that nutrition education and support groups significantly increase the adoption of orange-fleshed sweet potatoes. Specifically, participation in mother-to-mother nutrition support clubs and nutrition-focused health talks affects their adoption and diffusion, albeit with varying degrees of importance.

In their study, Jada and van den Berg (37) found that nutritional information alone significantly increases Ethiopian farmers’ interest in biofortified maize seeds, while social norm messaging has a limited impact. Their study highlighted the importance of decentralized vine multipliers and social factors in promoting the adoption of biofortified crops. They concluded that practical farmer education and extension programs proved to be the most effective strategies for adoption.

Our hedonic analysis revealed that the Bayoda + Aziz blend had the highest overall liking scores. This finding aligns with that in the study by Cheung et al. (38), who found that replacing up to 20% of wheat with fermented and non-fermented pearl millet flour had no measurable effect on liking or purchase intent. Their findings indicated that there were no sensory barriers to the partial substitution of whole-grain pearl millet for whole-grain wheat in the United States’ consumers and food supply. Similarly, in their study, Alamu et al. (39) found that blending biofortified yellow-endosperm maize with soybean flour enhanced carotenoid and protein content and improved functional qualities. However, it reduced the bulk density, dispersibility, swelling power, and pasting viscosity, while increasing solubility. These findings suggest that blending biofortified cultivars with local favorites offers a promising short-term strategy to overcome sensory resistance while delivering nutritional benefits. From a market perspective, understanding sensory profiles enables breeders and food processors to tailor products to segmented consumer preferences. This study provides a sensory-based foundation for positioning biofortified products in markets such as school feeding programs, health clinics, or maternal nutrition interventions, where sensory familiarity matters as much as nutrient content.

This study opens avenues for further research on the optimal blending strategies for Aziz and Sudanese pearl millet cultivars in *Aceda* preparation. Future investigations should explore the physicochemical, functional, thermal, and pasting properties of biofortified *Aceda* and their links to sensory attributes. Advanced sensory technologies, such as electronic noses (e-noses) and electronic tongues (e-tongues), can be employed to analyze flavor and aroma profiles, thereby enhancing commercialization. According to a study by Ma et al. (40), combining the e-nose and e-tongue provides a rapid and accurate method for evaluating instant biofortified pearl millet *Aceda*, aiding product development and consumer acceptance. In addition, artificial intelligence (AI)-driven sensory evaluation tools and machine learning algorithms now offer opportunities to predict consumer liking scores based on physicochemical traits and high-throughput metabolomic data. Integrating these tools into biofortification breeding pipelines could drastically reduce the cost and subjectivity of sensory trials, enabling faster scaling and targeting of biofortified products to diverse consumer groups.

Our internal preference mapping (IPM) and clustering analysis (Figures 4, 5) emphasize the importance of market-oriented breeding strategies that integrate sensory evaluation early in the pearl millet breeding pipeline. Consumer preferences varied, with some favoring traditional cultivars, such as Ashana, while others preferred the Bayoda + Aziz blend, which includes biofortified cultivars. This supports the findings reported in the study by Huey et al. (41), who emphasized that biofortification breeding enhances market appeal through preferred sensory attributes.

As pearl millet gains recognition for its nutritional benefits and climate resilience, biofortification must integrate indigenous food-processing knowledge with modern omics techniques. This is particularly critical in Sudan, where conflict-driven food insecurity has placed over half of the population at risk of acute malnutrition. Therefore, collaborative efforts among plant breeders, food technologists, and local communities should leverage Sudan’s pearl millet heritage while adopting advanced breeding techniques to enhance the acceptance and impact of food security.

The panel analysis showed that firmness, color, and appearance had the highest discriminative power, indicating that consumers subconsciously relied on taste, visual, and textural cues when rating *Aceda* (Table 2). PLSR and VIP analysis (Figures 10, 11) further identified taste, firmness, aroma, and texture as key drivers of consumer acceptance (Table 2). These findings suggest that breeding efforts should integrate sensory attributes, nutritional values, and market appeal (14, 41). Targeting these key descriptors using multiple sensor technologies and multivariate analysis can guide pearl millet breeders in developing new market-preferred varieties. However, comprehensive sensory and consumer research programs incorporating direct consumer feedback remain underdeveloped in Sudan’s pearl millet breeding program.

Agglomerative hierarchical clustering (AHC) analysis (Figure 6) grouped assessors into four distinct clusters based on their preferences. Cluster 1 disliked Ashana but preferred blended *Aceda*, while cluster 2 favored Ashana alone. Cluster 3 preferred blended *Aceda* products but rejected Aziz and Bayoda individually, whereas cluster 4 disliked Aziz but accepted the Bayoda + Aziz blend. These findings highlight that no single *Aceda* product can satisfy all consumer segments, reinforcing the need for market segmentation and targeted breeding. This aligns with the findings reported in the study by Arnaud et al. (42), who emphasized the importance of consumer preferences, food quality, and market-driven breeding of root, tuber, and banana crops. Similarly, Dufour et al. (43) highlighted the role of a comprehensive market analysis in aligning variety development with user expectations along the crop value chain. To address this, multidisciplinary teams should integrate sensory evaluation into pearl millet breeding to ensure that food product qualities align with Sudanese consumer preferences. This approach bridges the gap between production and consumption, enhancing the relevance of biofortified pearl millet-based products and market success in Sudan and beyond.

A gender-based clustering analysis (Figure 7) revealed distinct sensory preferences between men and women. Women predominantly in cluster 3 preferred blended *Aceda* products, whereas those in cluster 2 favored Ashana alone. In contrast, men were primarily presented in cluster 4, showing a preference for Bayoda + Aziz, while disliking Aziz. The absence of males in cluster 3 indicated a clear divide in taste perception. This finding supports the notion of Thiele et al. (44), who highlighted in their study that the importance of understanding and responding to gender differences in consumer preferences for food quality and post-harvest traits, which will contribute to the description of product profiles. The same authors have noted that women tend to prefer banana cultivars intended for household consumption. At the same time, men were more likely to use hybrids because of their suitability for the brewing industry. Similarly, in a study, Wossen et al. (45) found that women prefer cassava to be peeled easily. Such gendered preferences should be proactively addressed by pearl millet breeding programs through gender-disaggregated trials and inclusive product design. More research is needed to understand and address gender differences in consumer preferences for biofortified pearl millet-based products, which requires broader breeding program methods to capture and effectively utilize gender-differentiated information (46).

Integrating sensory evaluation into breeding programs for biofortified pearl millet is essential to enhance consumer acceptance and market adoption. Policies should promote multidisciplinary collaboration, blending biofortified with traditional cultivars and utilizing advanced sensory technologies. Market segmentation should be implemented to address diverse consumer preferences across different socioeconomic, cultural, and gender groups. Capacity building in sensory methods and continuous community engagement will align breeding decisions closely with market needs, ultimately facilitating successful commercialization, improving food security, and maximizing nutritional impact.

## 5 Conclusion

This study highlights that nutritional enhancement alone is insufficient for consumer acceptance of biofortified pearl millet products. *Aceda* made solely from the biofortified cultivar Aziz scored lowest in sensory evaluations. At the same time, the Bayoda + Aziz blend was preferred, emphasizing the importance of integrating taste, firmness, aroma, and appearance into biofortification breeding. We recommend incorporating early-stage sensory screening, local taste preferences, gender-sensitive selection, and AI-enabled sensory analytics into breeding programs to enhance product quality and consumer satisfaction. These strategies can accelerate the development of nutritious, consumer-preferred products, particularly in climate-vulnerable regions such as Sudan. In conclusion, aligning sensory quality with nutritional goals is essential for the successful adoption of biofortified crops. By combining traditional and biofortified cultivars and using advanced sensory tools, breeders can improve market acceptance and address malnutrition more effectively.

## Data Availability

The raw data supporting the conclusions of this article will be made available by the authors, without undue reservation.
